# Assessment of left atrial volume before and after pulmonary thromboendarterectomy in chronic thromboembolic pulmonary hypertension

**DOI:** 10.1186/1476-7120-12-32

**Published:** 2014-08-11

**Authors:** Nicholas A Marston, William R Auger, Michael M Madani, Bruce J Kimura, G Monet Strachan, Ajit B Raisinghani, Anthony N DeMaria, Daniel G Blanchard

**Affiliations:** 1 School of Medicine and Sulpizio Cardiovascular Center, University of California San Diego, 9444 Medical Center Drive, #7411, La Jolla, CA 92037, USA; 2 Scripps Health, 501 Washington St. Ste 512, San Diego, CA 92103, USA

**Keywords:** Left atrial volume, Chronic thromboembolic pulmonary hypertension, Pulmonary thromboendarterectomy

## Abstract

**Background:**

Impaired left ventricular diastolic filling is common in chronic thromboembolic pulmonary hypertension (CTEPH), and recent studies support left ventricular underfilling as a cause. To investigate this further, we assessed left atrial volume index (LAVI) in patients with CTEPH before and after pulmonary thromboendarterectomy (PTE).

**Methods:**

Forty-eight consecutive CTEPH patients had pre- & post-PTE echocardiograms and right heart catheterizations. Parameters included mean pulmonary artery pressure (mPAP), pulmonary vascular resistance (PVR), cardiac index, LAVI, & mitral E/A ratio. Echocardiograms were performed 6 ± 3 days pre-PTE and 10 ± 4 days post-PTE. Regression analyses compared pre- and post-PTE LAVI with other parameters.

**Results:**

Pre-op LAVI (mean 19.0 ± 7 mL/m^2^) correlated significantly with pre-op PVR (R = -0.45, p = 0.001), mPAP (R = -0.28, p = 0.05) and cardiac index (R = 0.38, p = 0.006). Post-PTE, LAVI increased by 18% to 22.4 ± 7 mL/m^2^ (p = 0.003). This change correlated with change in PVR (765 to 311 dyne-s/cm^5^, p = 0.01), cardiac index (2.6 to 3.2 L/min/m^2^, p = 0.02), and E/A (.95 to 1.44, p = 0.002).

**Conclusion:**

In CTEPH, smaller LAVI is associated with lower cardiac output, higher mPAP, and higher PVR. LAVI increases by ~20% after PTE, and this change correlates with changes in PVR and mitral E/A. The rapid increase in LAVI supports the concept that left ventricular diastolic impairment and low E/A pre-PTE are due to left heart underfilling rather than inherent left ventricular diastolic dysfunction.

## Background

Chronic thromboembolic pulmonary hypertension (CTEPH), the result of unresolved thrombus in the pulmonary vasculature following pulmonary embolism, carries a high rate of morbidity and mortality if left untreated [1-3]. In addition to the well-described right-sided dysfunction seen in CTEPH, left ventricular (LV) diastolic impairment is also common. Diminished early diastolic filling of the LV is frequently present, resulting in reversal of the “E” (early rapid filling) and “A” (atrial kick) velocities in mitral inflow.

Several theories have been put forth to explain this LV diastolic “dysfunction”, including decreased LV volume and compliance due to leftward septal shifting, and true intrinsic dysfunction of the LV [4]. More recent studies, however, have supported the theory of decreased RV output and relative LV underfilling as the primary cause of the perceived LV diastolic impairment in CTEPH [5-7]. If this is correct, one might hypothesize that left atrial volume would increase after successful pulmonary thromboendarterectomy (PTE) and potentially correlate with improvements in right heart catheterization measurements. We investigated this in a relatively large group of patients with CTEPH referred for PTE at our institution.

## Methods

### Patient population

This is a retrospective analysis of forty-eight consecutive CTEPH patients undergoing PTE at a large CTEPH referral center. The study cohort was comprised of 24 men and 24 women with a mean age of 52 ± 16 years, ranging from 19 to 84. All patients had class III to IV symptoms according to the New York Heart Association (NYHA) Functional Classification. Pre- and postoperative echocardiograms and right heart catheterizations were performed in each case. All PTE surgeries were completed at UCSD Medical Center using methods outlined in previous publications [8]. The UCSD institutional research review committee approved the study.

### Echocardiography

Echocardiograms were performed 6 ± 3 days before and 10 ± 4 days following PTE. A Vivid cardiovascular ultrasound system (GE VingMed, Horton, Norway) was used for all cases. Studies included measurements of the left atrial volume (LAVI), mitral E/A ratio, and mitral annular E/E’ ratio. Measurements of the left ventricle were also acquired, including cardiac index (CI) and end-systolic and end-diastolic diameters. All echocardiographic techniques followed the recommendations of the American Society of Echocardiography [9]. Maximum left atrial volume was measured at end-systole and then indexed using body surface area, a technique that has demonstrated the strongest correlation to cardiovascular risk stratification [10].

### Right heart catheterization

Right heart catheterization (RHC) using a Swan-Ganz catheter was performed within 48 hours of the pre-operative echocardiogram (6 ± 5 days prior to surgery) and on post-operative day 1. Invasive measurements included mean pulmonary artery pressures (mPAP), pulmonary vascular resistance (PVR), pulmonary capillary wedge pressure (PCWP), and cardiac output. All pressure measurements were obtained at end-expiration; three measurements were recorded for each variable and then averaged for a final value. Cardiac output was calculated using thermodilution; again, three measurements were obtained and averaged for a final value. PVR was calculated using the formula: PVR = 80(mPAP–mPCWP)/cardiac output.

### Statistical analysis

Pre- and post-operative values are expressed as mean ± standard deviation. Differences in left atrial volume and other characteristics before and after PTE were evaluated using a two-tailed paired Student’s t-test. A p value <0.05 was considered statistically significant. Linear regression analyses were performed to determine relationships between LA volume and other cardiac parameters using online regression software (http://www.wessa.net) [11].

## Results

Prior to PTE all patients had evidence of significantly elevated right-sided pressures [mPAP of 45.5 ± 9.6 mmHg and PVR of 765 ± 396 (dyne-sec)/cm^5^], and many had depressed cardiac output (mean CO of 3.9 ± 1.2 l/min). Evidence of diastolic dysfunction was also present, with decreased mitral E/A ratio (0.95 ± 0.31) and increased deceleration time (224 ± 60 ms). Mitral lateral annular E/E’ was 6.1 ± 2.8. Of note, pre-operative left atrial volume index was relatively low (19.0 ± 7.0 cm^3^/m^2^). All baseline hemodynamic and echocardiographic findings are listed in Table 1.

**Table 1:** Hemodynamic and echocardiographic characteristics pre- and post-PTE

	**Pre-PTE**	**Post-PTE**	**p value**
**Right heart catheterization**			
Mean PA pressure (mmHg)	45.5 ± 9.6	28.8 ± 6.7	<0.001
PVR (dyne-sec)/cm^5^	765 ± 396	310 ± 143	<0.001
Cardiac output (L/min)	3.9 ± 1.2	5.2 ± 1.6	<0.001
**Echocardiogram**			
Cardiac index	2.6 ± 0.8	3.2 ± 0.5	<0.001
Mitral E/A ratio	0.95 ± 0.31	1.45 ± 0.48	<0.001
Lateral E/E’ ratio	6.1 ± 2.8	7.9 ± 2.7	<0.001
Deceleration time (ms)	224 ± 60	189 ± 39	<0.001
Left atrial volume index (cm^3^/m^2^)	19.0 ± 7.0	22.4 ± 6.8	0.003

**Figure 1:**
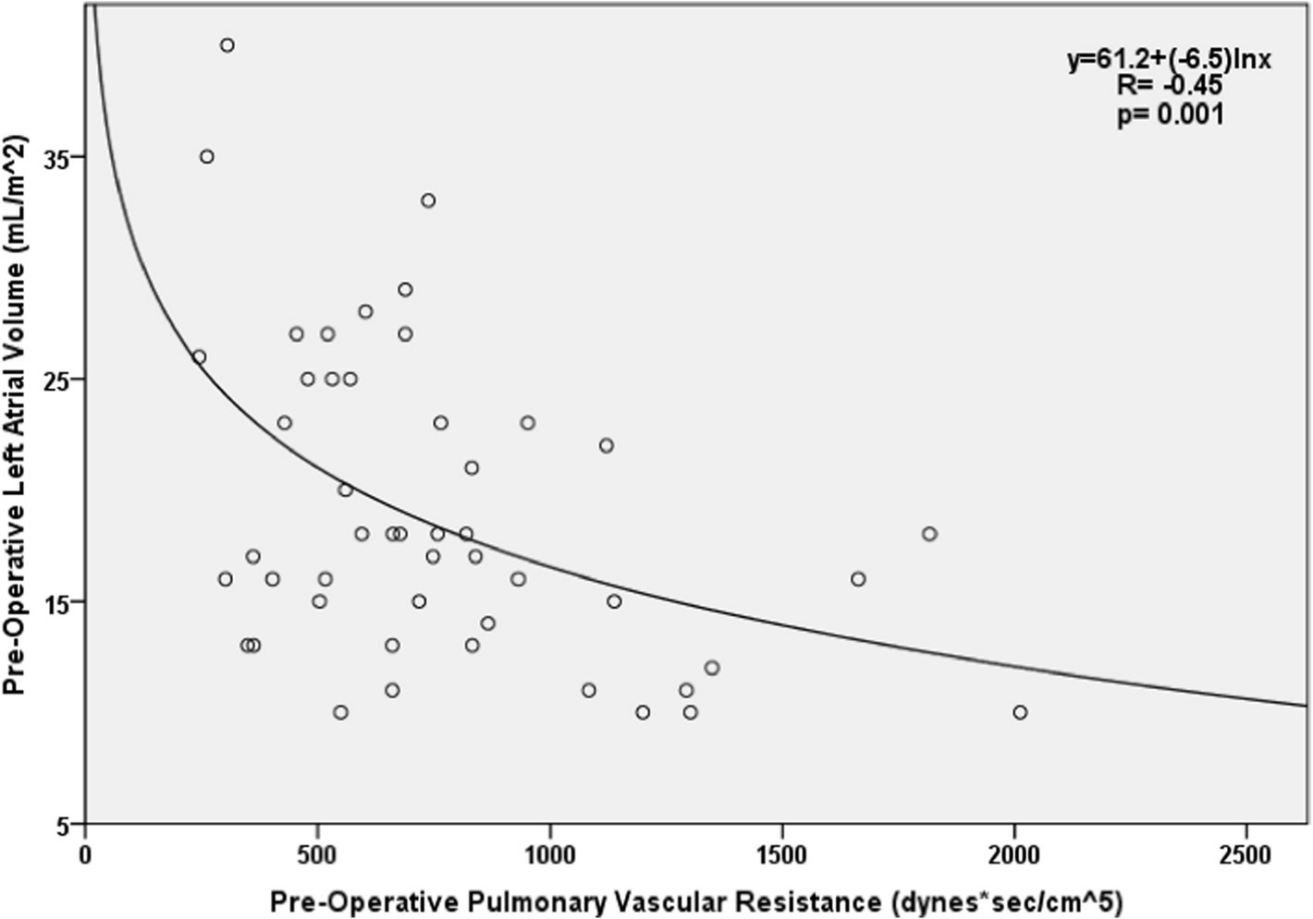
Regression analysis of pre-operative LA volume index vs. PVR.

Following surgery significant improvements in hemodynamics were observed. The mean pulmonary arterial pressure fell to 28.8 ± 6.7 mmHg (p < 0.001) and pulmonary vascular resistance dropped to 310 ± 143 (dyne-sec)/cm^5^ (p < 0.001). This normalization of right-sided pressures coincided with an improvement in cardiac output to 5.2 ± 1.6 L/min (p < 0.001). Further, LV diastolic function improved post-operatively, with the E/A ratio rising to 1.45 ± 0.48 (p < 0.001) and the E deceleration time dropping into the normal range (189 ± 39 ms) (p < 0.001). A significant increase in left atrial volume index also occurred (from 19.0 ± 7.0 to 22.4 ± 6.8 cm^3^/m^2^) (p = 0.003). Mitral annular E/E’ increased to 7.9 ± 2.7 (p < 0.001). The changes in each variable following surgery are listed in Table 1.Left atrial volume not only increased with surgery but also correlated well with established CTEPH parameters. In the pre-operative state, LA volume index was inversely correlated with PVR (R= -0.45, p = 0.001, Figure 1), and mPAP (R = -0.28, p = 0.05), and was positively correlated with cardiac index (R = 0.48, p = 0.001). Smaller pre-op LA volumes also correlated with markers of diastolic dysfunction, such as greater E deceleration times (R = -0.34, p = 0.009) and lower mitral annular E/E’ (R = 0.36, p = 0.03).

**Figure 2:**
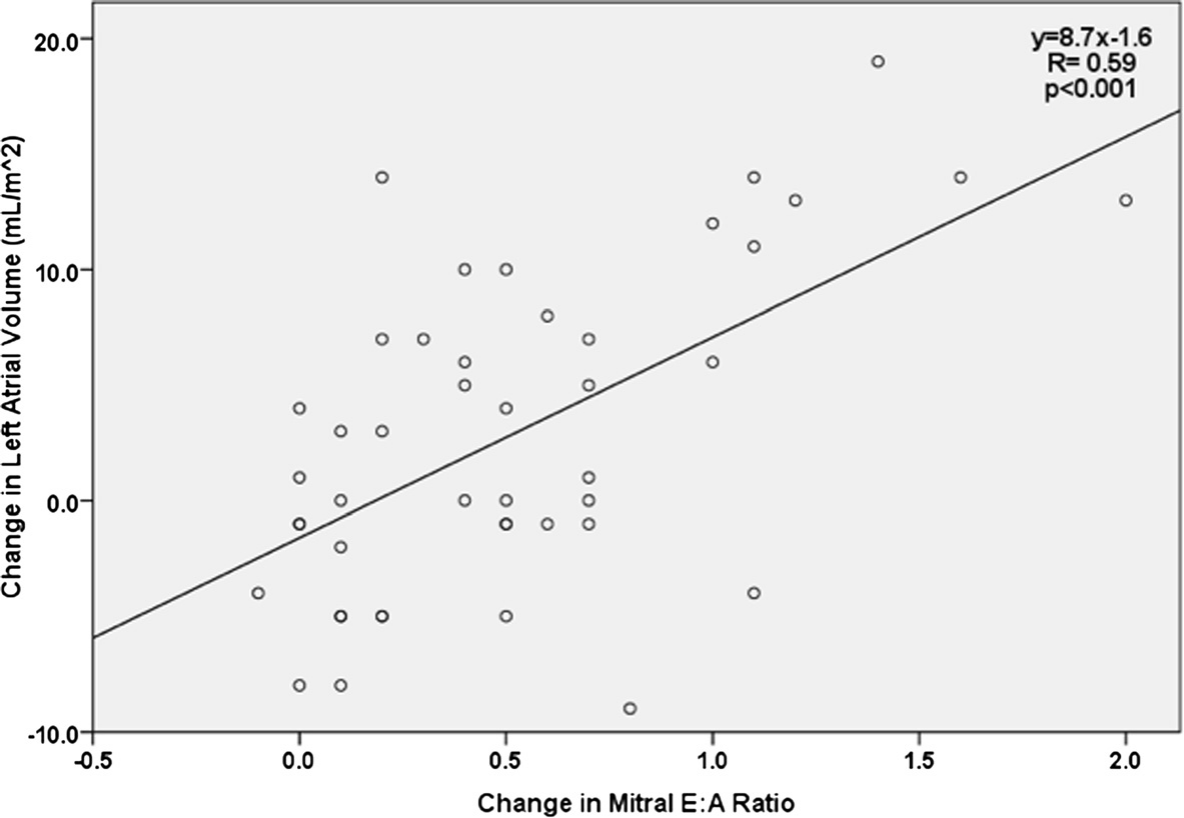
Regression analysis of change in LA volume vs. change in mitral E/A ratio.

Post-PTE, the change seen in LA volume index was associated with improvement in many of these same parameters. LA volume change correlated significantly with decrease in PVR (R= -0.36, p = 0.01) and increase in cardiac index (R= 0.41, p = 0.007). Similarly, increasing LA volume index correlated with improving diastolic function, such as an increase in mitral E/A ratio (R = 0.44, p < 0.001, Figure 2) and E/E’ (R = 0.36, p = 0.03). All correlation coefficients are listed in Table 2.

**Table 2:** Correlation coefficients and p values for left atrial volume vs. RHC and echo parameters

	**R**	**p value**
**Pre-PTE**		
LAVI vs PVR	-0.45	0.001
LAVI vs mPAP	-0.28	0.05
LAVI vs CO	0.39	0.006
LAVI vs CI	0.48	0.001
LAVI vs Mitral E/A ratio	0.13	0.41
LAVI vs E/E’ ratio	0.36	0.03
LAVI vs Decel time	-0.34	0.009
**Change after PTE**		
LAVI vs PVR	-0.36	0.01
LAVI vs mPAP	-0.06	0.68
LAVI vs CO	0.08	0.93
LAVI vs CI	0.41	0.007
LAVI vs Mitral E/A ratio	0.44	<0.001
LAVI vs E/E’ ratio	0.36	0.03
LAVI vs Decel time	-0.24	0.11

## Discussion

The relevance of left atrial volume and function in overall cardiovascular health has been increasingly recognized in the last several years. LA volume is now understood to be a noninvasive “biomarker” that can predict cardiovascular risk and prognosis in a wide range of cardiovascular diseases [12]. At a physiologic level, the contribution of the left atrium to the performance of the left ventricle is well established [13], but alterations in this relationship for different disease states remain an area of interest.

This study is the first to examine left atrial volume in patients with CTEPH before and after PTE. We demonstrated that LA volumes are in the low-normal range in this population, and that LAVI significantly increases following PTE. Furthermore, smaller LA volumes before surgery are a marker for disease severity, and are associated with higher PVR, higher mPAP, and lower CO. This suggests that LA volume may have a role in the evaluation of CTEPH patients prior to right heart catheterization and PTE. Additionally, the increase in LA volume following PTE is significantly correlated with improvement in PVR, providing another potential non-invasive marker for post-operative success.

Our findings also provide evidence regarding the origin of LV diastolic dysfunction in CTEPH. This has been an area of research interest since PTE was developed as the definitive therapy for CTEPH [14,15]. In 1989, Dittrich et al. demonstrated improvement in LV diastolic filling with relief of chronic RV pressure overload [16]. In 2002, Mahmud et al. used the E/A ratio to show that early diastolic filling is primarily impaired in CTEPH, and that early LV filling improves with successful PTE [5]. In 2007, Gurudevan et al. evaluated whether the abnormal diastolic filling pattern (E < A) often seen in CTEPH was due to an intrinsic LV abnormality or impaired LV filling. The results suggested that that the abnormal E/A pattern was in large part due to decreased LV preload [6]. These findings were supported by Lumens et al. using a computer-generated model that could separate the influences of septal bulging vs. LV filling on transmitral Doppler flow patterns pre- and post-PTE [7]. Gurudevan also reported that early diastolic mitral annular velocity (E’) was abnormally low in CTEPH but increased after PTE. As in the present study, Gurudevan also showed that mitral annular E/E’ rose significantly after PTE. Importantly, *both* E and E’ increased postoperatively, with a greater proportion of increase in the E velocity [6].

As these studies are consistent with decreased preload as the cause of LV diastolic impairment in CTEPH, we decided to focus on the characteristics of the left atrium in this population. In this study, we found that smaller pre-operative LA volumes were significantly correlated with longer E wave deceleration times (i.e., impaired early diastolic filling). Furthermore, the subsequent increase in LA volume following PTE was significantly correlated with higher mitral E/A ratio. Both of these findings support the theory of LV underfilling and suggest that LA volume is a significant component of the process.

Whether LA volume in CTEPH is limited solely by decreased LA filling or by anatomic restrictions as well is not entirely clear. A recent study suggests that changes in RV outflow tract dimension may affect left atrial filling and compliance in CTEPH [17]. A proposed explanation is that the mediastinal space between the sternum and the spine is relatively fixed: an increase in RV and RV outflow tract size may limit normal expansion of the left atrium. Another recent report documented compression of the LA and RV by large hiatal hernias within the mediastinum [18]. This concept was demonstrated “in reverse” in a dog model, where pericardiectomy led to an increase in LA compliance, reservoir function, and early diastolic LV filling [19].

### Limitations

A limitation in our study was the timing of the preoperative transthoracic echocardiogram and right heart catheterization. They were not performed simultaneously and in some instances up to 48 hours elapsed between procedures. As this population was stable preoperatively, it is unlikely this had a significant impact on the results. Post-operatively, echocardiography was performed an average of 9 days after PTE. It is conceivable that cardiac dimensions may have varied during this period, but again these patients were overall stable following surgery. As with several previous studies from our institution, echocardiography was delayed until patients left the surgical intensive care unit and could be examined safely in the noninvasive cardiac laboratory [5,6,20].

## Conclusions

This study was designed to evaluate LA volume characteristics in CTEPH patients undergoing PTE. This data suggest that LA volume is inversely associated with severity of CTEPH, and that LA volume increases after PTE. The rapid increase in LAV and correlation with improvement in E/A ratio supports the concept that left ventricular diastolic impairment is due primarily to left heart underfilling rather than inherent left ventricular diastolic dysfunction. The long-term prognostic implications of LA volume change after PTE deserves further study.

## Abbreviations

CTEPH: Chronic thromboembolic pulmonary hypertension; LV: Left ventricle; LA: Left atrium; PTE: Pulmonary thromboendarterectomy; NYHA: New York Heart Association; UCSD: University of California San Diego; LAVI: Left atrial volume index; CI: Cardiac index; RHC: Right heart catheterization; mPAP: Mean pulmonary artery pressure; PVR: Peripheral vascular resistance; PCWP: Pulmonary capillary wedge pressure; CO: Cardiac output.

## Competing interests

The authors declare that they have no competing interest.

## Authors’ contributions

All authors contributed to the study design, data collection, data analysis, and writing of the manuscript. All authors read and approved the final manuscript.

## Authors’ information

This manuscript originates from the Cardiovascular Center at the University of California, San Diego Medical Center, which has the highest-volume program for chronic thromboembolic pulmonary hypertension (CTEPH) and pulmonary thromboendarterectomy in the Western hemisphere. Dr. Blanchard has been senior author on a number of papers describing the interactions of the right and left ventricles in CTEPH over the last several years. Dr. Auger is a senior pulmonary specialist within the group, and Dr. Madani performs the majority of pulmonary thromboendarterectomies. Dr. Anthony DeMaria is the immediate past Editor-in-Chief of the Journal of the American College of Cardiology, and is a pre-eminent authority in echocardiography.
